# Associations between intakes of foods and their relations to overweight/obesity in 16-year-old adolescents

**DOI:** 10.1017/jns.2022.24

**Published:** 2022-04-07

**Authors:** Maria Norburg Tell, Katarina Hedin, Mats Nilsson, Marie Golsäter, Hans Lingfors

**Affiliations:** 1 Futurum, Region Jönköping County, Jönköping, Sweden; 2 Department of Health, Medicine and Caring Sciences, Linköping University, Linköping, Sweden; 3 Department of Clinical Sciences in Malmö, Family Medicine, Lund University, Malmö, Sweden; 4 Child Research Group, Department of Nursing, School of Health and Welfare, Jönköping University, Jönköping, Sweden

**Keywords:** Adolescents, Food intake, Health promotion, isoBMI, School Health Services, CI, confidence Interval, isoBMI, age- and sex-adjusted body mass index, NNR, Nordic Nutrition Recommendations, OR, odds ratio, SSBs, sugar-sweetened beverages

## Abstract

There is limited knowledge about the associations between intakes of different foods and inconsistency in the literature of the relation between the quality of food intake and bodyweight in adolescents. The aim of this study is to explore how healthy self-reported food intakes are associated with each other and with overweight/obesity in adolescents. This is a cross-sectional study of seven cohorts of adolescents (*n* 13 451) who turned sixteen from 2009/2010 up to 2015/2016 and responded to a health questionnaire used by the School Health Services in southeast Sweden. Associations between intakes of ten self-reported foods as well as between food intakes and weight groups based on the International Obesity Task Force standards (isoBMI) were explored by multivariable logistic regression. Healthy intakes of different foods were mostly associated with each other with the strongest association between a high intake of fruit and a high intake of vegetables (odds ratio (OR) = 25 (95 % confidence interval (CI) 20⋅0–33⋅1)). A low-frequency intake of sweets/snacks (OR = 2⋅35 (95 % CI 1⋅84–3⋅00)) was associated with overweight/obesity as well as a healthy choice of butter/margarine (≤40 % fat) (OR = 1⋅82 (95 % CI 1⋅39 to 2⋅41)), but a high-frequency intake of vegetables was negatively associated with overweight/obesity 0⋅77 (0⋅62–0⋅95). To promote health and achieve a healthy weight among adolescents, it is important to take both diet quality and total food amount into consideration.

## Introduction

Nutritious food is important for normal body function and growth as well as for the prevention of diseases^(1)^. Still few adolescents eat according to the national recommendations^(2)^. A recently published study showed a deterioration in terms of dietary quality during adolescence^(3)^. In order to find methods aiming to nudge towards a healthier food intake, it is of importance to explore if, and if so, how different foods associate with each other. Associations between intakes of different foods have previously been described in young children, but there is only scarce knowledge of such associations in adolescents^(4,5)^. It is likely that the food intake among small children mirrors their parents’. For adolescents, who have gained some autonomy, it is plausible that there will be influence from others than their families, which means that they may be open to new choices, and hence, interventions during adolescence are of interest^(6)^.

Key factors concerning body weight are the total energy intake and energy expenditure from physical activity. Concerning the association between weight and the quality of food, the field of knowledge is not so clear. Despite the sugar content, fruit intake in adults may have a reverse effect on obesity due to high water and fibre content, as well as high inherent of micronutrients and phytochemicals, which might affect modulation in gut ecology^(7)^. One systematic review stated that a high intake of vegetables had a reverse effect on weight in adults^(8)^. On the other hand, another review concluded that both a high intake of healthy food and a high intake of unhealthy food were associated with higher body mass index (BMI) among adolescents^(9)^. This shows that the relationship between body weight and the quality of food is unclear.

Due to the scarce knowledge of associations between different foods in adolescents and the inconsistent association between body weight and reported intake of different foods, it is of great importance to increase knowledge about associations between intakes of healthy as well as of unhealthy foods in adolescents and their associations to overweight and obesity. The aim of this study is to explore how different healthy self-reported food intakes are associated with each other and with overweight/obesity in adolescents.

## Methods

### Setting

In southeast Sweden in Jönköping County, a questionnaire, called ‘My Health’^(10)^, is used as a basis for a dialogue between a school nurse and a 16-year-old adolescent at health visits at the School Health Services. A health visit should be offered during upper secondary school according to the Swedish Education Act^(11)^. The questions in the questionnaire were based on a larger validated questionnaire for adults^(12)^ and further developed to be useful in health dialogues^(10)^. Before the health visit, the adolescents respond to the questionnaire in the classroom, with support from the school nurse. During the health visit, the school nurse measures the adolescent without shoes and incorporates the measurements in the paper questionnaire. Height is measured to the nearest centimetre (cm) in metre (m) and weight, in light clothing, to the nearest 100  gram (g) in kilogram (kg). The information from these questionnaires was then recorded into a digital database at Futurum, Region Jönköping County. Between 2009/2010 and 2015/2016 schools, corresponding to approximately half of the 16-year-old adolescents in Jönköping County used the questionnaire.

### Study population

This was a cross-sectional study of seven cohorts of adolescents who turned sixteen during the academic years 2009/2010 up to 2015/2016 from upper secondary schools who participated in a health visit and answered the ‘My Health’ questionnaire. The total number of participants during the period was 13 451 adolescents. No exclusion criteria were used.

### Data collection

In the present study, data from questions about self-reported food intakes (fish, vegetables, fruit, mealtime beverages, butter/margarine as a sandwich spread, sandwich toppings, juice/chocolate drinks, sugar-sweetened beverages (SSBs), sweets/snacks and pastries) and the measurements of body weight and body height were extracted from the database. For seven out of ten questions concerning food intake, the adolescents stated the frequency of their consumption during the past 7 d. There were four to seven possible response alternatives. For three of the questions, the adolescents just stated what type of food they used to consume (Table 1).

**Table 1.** Reported intake of different foods in 16-year-old adolescents according to the ‘My Health’ questionnaire

Questions If you think of the past 7 d …	Response alternatives	Classification of response alternatives	Adolescents *n* 13 451 *n* (%)^a^
… how often did you have fish	At least 3 times/week	Healthy	827 (6)
	Twice/week		4419 (33)
	Once/week	Moderately healthy	5710 (43)
	Never	Unhealthy	2468 (18)
… how often did you have vegetables?	At least 3 times/day	Healthy	770 (6)
	About twice/day		3402 (25)
	About once/day	Moderately healthy	4107 (31)
	5–6 times/week		1437 (11)
	3–4 times/week		1797 (13)
	1–2 times/week	Unhealthy	1431 (11)
	Never		491 (4)
… how many fruits did you have?	At least 3 fruits/day	Healthy	666 (5)
	About 2 fruits/day		1854 (14)
	About 1 fruit/day	Moderately healthy	3284 (24)
	About 3–4 fruits/week		2880 (21)
	About 1–2 fruits/week	Unhealthy	3237 (24)
	No fruit		1513 (11)
… what mealtime beverages did you usually have?	Water	Healthy	5585 (42)
	Milk (0⋅5 % fat)		1624 (12)
	Milk (1⋅5 % fat)	Moderately healthy	4048 (30)
	Milk (3⋅0 % fat)	Unhealthy	855 (6)
	Soda or juice		1316 (10)
… what butter/margarine as a sandwich spread did you usually have?	None	Healthy	999 (7)
	30–40 % fat		7066 (53)
	60 % fat	Moderately healthy	3268 (24)
	80 % fat	Unhealthy	1948 (15)
… what sandwich toppings did you usually have?	None	Healthy	1222 (9)
	Fruits or vegetables		894 (7)
	Ham, turkey, egg, low-fat cheese, mackerel or caviar		9743 (72)
	Marmalade or honey	Unhealthy	460 (3)
	Sausage or high-fat cheese		1020 (8)
… how often did you have at least one glass of chocolate drink or juice?	Never	Healthy	3292 (25)
	Once/week		2914 (22)
	2–3 times/week	Moderately healthy	3764 (28)
	4–5 times/week	Unhealthy	1578 (12)
	Almost every day		1564 (12)
	Several times/day		310 (2)
… how often did you have at least one glass of soft drink, energy/sports drink? (sugar-sweetened beverages)	Never	Healthy	1524 (11)
	Once/week		2896 (22)
	2–3 times/week	Moderately healthy	5646 (42)
	4–5 times/week	Unhealthy	2050 (15)
	Almost every day		965 (7)
	Several times/day		342 (3)
… how often did you have sweets or snacks (chocolate, ice cream, sweets and chips)?	Never	Healthy	995 (7)
	Once/week		3560 (27)
	2–3 times/week	Moderately healthy	6670 (50)
	4–5 times/week	Unhealthy	1501 (11)
	Almost every day		591 (4)
	Several times/day		114 (0⋅8)
… how often did you have cookies, biscuits, buns or cake? (pastries)	Never	Healthy	3116 (23)
	Once/week		4896 (36)
	2–3 times/week	Moderately healthy	4140 (31)
	4–5 times/week	Unhealthy	885 (7)
	Almost every day		329 (2)
	Several times/day		60 (0⋅4)

The response alternatives presented (healthy, moderately healthy and unhealthy intake) are based on the NNR.

a Missing data were less than 1 % for all questions.

### Statistical methods

In the present study, the response alternatives were grouped into three categories: healthy, moderately healthy and unhealthy intakes (Table 1), based on dietary advice from the Swedish Food Agency and the Nordic Nutrition Recommendations (NNR)^(1,13)^. Descriptive results are presented as numbers and proportions.

One way to estimate body size is to use BMI which is a calculation of a person's weight (kg) divided by the square of height (m). The distribution of isoBMI categories for the adolescents, classified in age- and gender-specific BMI (isoBMI) according to The International Obesity Task Force (IOTF), is presented as numbers and proportions (Table 2)^(14,15)^. In the present study, two bodyweight groups were formed for the analysis: normal weight including thinness grade 1 (corresponding to adult BMI from 17 to 24⋅9) and overweight/obesity (corresponding to adult BMI ≥25), respectively. Adolescents with thinness grade 2 and grade 3 (corresponding to adult BMI <17) were few (1⋅2 %) and were excluded from further analysis.

**Table 2.** IsoBMI categories in 16-year-old adolescents 2009/2010–2015/2016 in Jönköping County

Distribution of isoBMI categories *n* 13 451 (5491 girls and 6366 boys)							
Gender	Thinness grade 3 (<16)	Thinness grade 2 (16–16⋅9)	Thinness grade 1 (17–18⋅4)	Normal weight (18⋅5–24⋅9)	Overweight (25⋅0–29⋅9)	Obesity (≥30)	Missing data isoBMI
Girls	31	88	492	3728	904	248	757
Boys	6	29	569	4004	1259	499	787
Missing data	0	2	0	36	5	0	7
Total	37	119	1061	7768	2168	747	1551

To examine the associations between intakes of different foods, data were analysed with uni- (not reported) and multivariable logistic regression. In step one, a univariable logistic regression was performed to evaluate explanatory variables to be included in the multivariable model. All explanatory variables in the univariable logistic regression (step one) with a *P*-value of ≤0⋅2 were included in the first multivariable analysis (step two). This is a recommended way for model building in order to minimise the risk of omitting variables that might contribute to the model building process^(16)^.

In step three, all variables that had a *P*-value of <0⋅05 in the first multivariable analysis (step two) were included in the semi-final model. In step four (final model), all variables in the semi-final model (step three) with a *P*-value of <0⋅05 were included in the final run of the model (step four). The resulting *P*-values for the included explanatory variables in step four were adjusted for multiple comparisons with Hochberg's method^(17)^, and all explanatory variables with a *P*-value of <0⋅05 after adjustment were kept in the final model. These steps were used to avoid ‘fishing’ for significance and give better control over the model building process^(18)^.

A logistic model was also used to estimate the probability of a healthy *v.* an unhealthy intake. The explanatory variables are coded into three categories, such as ‘healthy intake’, ‘moderately healthy intake’ and ‘unhealthy intake’. No interaction effects were assumed in the models. Interaction effects were tested for some explanatory variables but were not found.

The odds ratios (OR) for the reference level and the moderately healthy intake are not presented in the results (Table 3).

**Table 3.** Associations between reported intake of foods in 16-year-old adolescents (*n* 13 451) in Jönköping County from 2009/2010 up to 2015/2016 explored by multivariable logistic regression

	Healthy intake of foods
	Odds ratio (95 % CI)
	Fish *n* 7598
Vegetables	5⋅4 (4⋅6–6⋅4)
Fruit	2⋅2 (1⋅8–2⋅5)
Butter/margarine as a sandwich spread	0⋅8 (0⋅7–1⋅0)
Sugar-sweetened beverages	1⋅3 (1⋅1–1⋅5)
Sweets/snacks	1⋅3 (1⋅1–1⋅6)
Pastries	0⋅7 (0⋅6–0⋅8)
	Vegetables *n* 5958
Fish	5⋅6 (4⋅7–6⋅8)
Fruit	18⋅1 (14⋅7–22⋅3)
Mealtime beverages	2⋅3 (1⋅9–2⋅7)
Butter/margarine as a sandwich spread	0⋅7 (0⋅6–0⋅8)
Sandwich toppings	1⋅7 (1⋅3–2⋅0)
Sugar-sweetened beverages	2⋅0 (1⋅7–2⋅5)
Sweets/snacks	1⋅4 (1⋅2–1⋅8)
	Fruits *n* 4018
Fish	2⋅7 (2⋅2–3⋅4)
Vegetable	25⋅7 (20⋅0–33⋅1)
Mealtime beverages	1⋅4 (1⋅2–1⋅7)
Sugar-sweetened beverages	2⋅7 (2⋅2–3⋅3)
	Mealtime beverages *n* 9182
Vegetables	2⋅4 (2⋅0–2⋅8)
Fruits	1⋅4 (1⋅2–1⋅6)
Butter/margarine as a sandwich spread	1⋅5 (1⋅3–1⋅7)
Sandwich toppings	1⋅6 (1⋅4–1⋅9)
Juice/chocolate drinks	2⋅0 (1⋅7–2⋅2)
Sugar-sweetened beverages	5⋅0 (4⋅3–5⋅8)
Pastries	1⋅3 (1⋅1–1⋅5)
	Butter/margarine as a sandwich spread *n* 9938
Vegetables	0⋅7 (0⋅6–0⋅8)
Mealtime beverages	1⋅6 (1⋅4–1⋅9)
Sandwich toppings	1⋅2 (1⋅0–1⋅4)
Sugar-sweetened beverages	0⋅7 (0⋅6–0⋅8)
Sweets/snacks	1⋅3 (1⋅1–1⋅6)
Pastries	1⋅3 (1⋅1–1⋅5)
	Sandwich toppings *n* 13 296
Vegetables	1⋅7 (1⋅4–2⋅0)
Mealtime beverages	1⋅6 (1⋅4–1⋅9)
Juice/chocolate drinks	1⋅3 (1⋅1–1⋅5)
Sweets/snacks	1⋅2 (1⋅1–1⋅5)
Pastries	1⋅5 (1⋅3–1⋅8)
	Juice/chocolate drinks *n* 9543
Vegetables	1⋅2 (1⋅1–1⋅4)
Mealtime beverages	2⋅0 (1⋅8–2⋅2)
Sandwich toppings	1⋅3 (1⋅1–1⋅5)
Sugar-sweetened beverages	2⋅3 (2⋅1–2⋅7)
Sweets/snacks	2⋅2 (1⋅9–2⋅5)
Pastries	1⋅7 (1⋅5–2⋅0)
	Sugar-sweetened beverages *n* 7651
Fish	1⋅3 (1⋅1–1⋅5)
Vegetables	2⋅1 (1⋅7–2⋅5)
Fruits	2⋅3 (2⋅0–2⋅8)
Mealtime beverages	4⋅2 (3⋅7–4⋅9)
Butter/margarine as a sandwich spread	0⋅6 (0⋅5–0⋅7)
Juice/chocolate drinks	2⋅5 (2⋅2–2⋅8)
Sweets/snacks	10⋅6 (9⋅0–12⋅5)
Pastries	1⋅7 (1⋅4–2⋅0)
	Sweets/snacks *n* 6611
Fish	1⋅3 (1⋅1–1⋅6)
Vegetables	1⋅4 (1⋅2–1⋅8)
Fruits	1⋅4 (1⋅1–1⋅7)
Butter/margarine as a sandwich spread	1⋅3 (1⋅1–1⋅6)
Sandwich toppings	1⋅2 (1⋅0–1⋅5)
Juice/chocolate drinks	2⋅0 (1⋅7–2⋅3)
Sugar-sweetened beverages	10⋅1 (8⋅5–12⋅0)
Pastries	9⋅2 (7⋅7–11⋅1)
	Pastries *n* 9087
Fish	0⋅8 (0⋅6–0⋅9)
Fruits	0⋅7 (0⋅6–0⋅9)
Mealtime beverages	1⋅4 (1⋅1–1⋅6)
Butter/margarine as a sandwich spread	1⋅3 (1⋅1–1⋅5)
Sandwich toppings	1⋅5 (1⋅2–1⋅8)
Juice/chocolate drinks	1⋅6 (1⋅4–1⋅9)
Sugar-sweetened beverages	1⋅8 (1⋅5–2⋅2)
Sweets/snacks	12⋅0 (9⋅8–14⋅8)

To examine the probability of overweight/obesity *v.* normal weight for different intakes of foods, multivariable logistic regression was performed with unhealthy intake (reference level) and healthy intake as explanatory variables (Table 4). These analyses were performed without taking other possible confounding variables, such as physical activity into account. The results from all the logistic regressions are presented as OR with ninety-five percent Confidence Intervals (95 % CI).

**Table 4.** Associations between healthy intakes of foods and overweight/obesity in 16-year-old adolescents (*n* 13 451^a^) in Jönköping County from 2009/2010 up to 2015/2016 explored by multivariable logistic regression

Healthy intake of foods	Overweight/obesity (isoBMI ≥ 25)	
	Crude odds ratio (95 % CI)	Odds ratio from final multivariable logistic regression^b^ (95 % CI)
Fish *n* 4681	0⋅91 (0⋅81–1⋅02)	Not in the final model
Vegetables *n* 4681	0⋅85 (0⋅75–0⋅98)	0⋅77 (0⋅62–0⋅95)
Fruits *n* 2919	0⋅98 (0⋅84–1⋅14)	Not in the final model
Mealtime beverages *n* 6214	1⋅53 (1⋅35–1⋅73)	Not in the final model
Margarine/butter as a sandwich spread *n* 7083	1⋅62 (1⋅42–1⋅85)	1⋅82 (1⋅39–2⋅41)
Sandwich toppings *n* 10 350	1⋅27 (0⋅98–1⋅29)	Not in the final model
Juice/drink of chocolate *n* 5361	1⋅70 (1⋅53–1⋅90)	Not in the final model
Sugar-sweetened beverages *n* 3841	1⋅05 (0⋅94–1⋅18)	Not in the final model
Sweets/snacks *n* 3937	2⋅29 (1⋅99–2⋅64)	2⋅35 (1.84–3⋅00)
Pastries *n* 6888	1⋅94 (1⋅65–2⋅29)	Not in the final model

a Frequency missing data for isoBMI *n* 1551, missing data for gender *n* 50.

b In the final model, 2048 adolescents were included in the calculations.

Statistica version 13 was used for the descriptive part of the analysis. SAS^®^ Stat version 15.2 software, Proc Logistic (Copyright© 2020 by SAS Institute Inc., Cary, NC, USA), was used for the logistic regression data analysis.

### Ethical approval

This study was conducted according to the guidelines in the Declaration of Helsinki, and all procedures involving human subjects were approved by the Regional Ethical Review Board in Linköping University, Sweden (Dnr 2018/19-31).

## Results

The reported intakes of different foods classified as healthy, moderately healthy and unhealthy intakes among the 13 451 adolescents (6248 girls, 7153 boys, 50 missing data concerning gender) are presented in Table 1.

A healthy choice (Table 1) of food intake was reported by 88 % of the participants for sandwich toppings, by 60 % for butter/margarine as a sandwich spread and by 54 % for mealtime beverages. A high-frequency intake was reported by 39 % for fish, by 31 % for vegetables and by 19 % for fruits. A low-frequency intake of unhealthy foods was reported by 60 % for pastries, by 46 % for juice/chocolate drinks, by 34 % for sweets/snacks and by 33 % for SSBs.

The distribution of adolescents in different isoBMI categories is presented in Table 2. Of the 11 900 adolescents with data for isoBMI, 74⋅2 % were classified as normal weight (including thinness grade 1) and 24⋅5 % as overweight/obese (1⋅2 % as thinness grade 2 and grade 3).

Most healthy foods were associated with other healthy foods (Table 3). A high-frequency intake of fruit was mainly associated with a high-frequency intake of vegetables, but also with a high-frequency intake of fish and a low-frequency intake of juice/chocolate drinks and SSBs. The strongest association was seen between a high-frequency intake of fruits and a high-frequency intake of vegetables with OR = 25 (95 % CI 20⋅0–33⋅1).

A low-frequency intake of SSBs was found to be associated with a low-frequency intake of other unhealthy foods, for example, a low-frequency intake of sweets/snacks and pastries, and with a high-frequency intake of healthy foods (Table 3).

The variation in outcome was explained by 40–43 % by variables such as fruits, vegetables, SSBs and sweets/snacks. The variations explained for butter/margarine as a sandwich spread and sandwich toppings were 2 and 3 %. For the rest of the outcome variables, the variations explained were between 13 and 20 % (Table 3).

Healthy and unhealthy intakes of foods for different isoBMI categories are presented in Table 4. A high-frequency intake of vegetables was found to be statistically significantly associated with lower odds for overweight/obesity. No association was found between a high-frequency intake of fish or fruit and isoBMI categories. Statistically significant associations were seen between overweight/obesity and a healthy choice of butter/margarine as a sandwich spread, and for a low-frequency intake of sweets/snacks. If gender was included in the multivariable logistic regression, no one of the food groups was statistically significant in the model. Girls had approximately 30 % lower risk for overweight/obesity compared to boys.

## Discussion

### Key results

In this cross-sectional study among 16-year-old adolescents, healthy intakes of different foods were mostly associated with each other with the strongest association between a high-frequency intake of fruits and a high-frequency intake of vegetables. A low-frequency intake of sweets/snacks and a healthy choice of butter/margarine were associated with overweight/obesity, but a high-frequency intake of vegetables was negatively associated with overweight/obesity.

### Interpretation

To the best of our knowledge, this is one of the few studies analysing associations between foods in 16-year-old adolescents and the first in European adolescents. Similar associations between healthy food choices were seen in Danish infants^(4)^ and Brazilian adolescents^(19)^.

There has been a trend in Sweden during the latest decade to eat butter instead of low-fat margarine, and this phenomenon has been perceived as healthy in some groups^(20)^. This might partly explain the present results where intake of unhealthy butter/margarine as a sandwich spread (high-fat percentage and high proportion of saturated fat) was weakly associated with intake of healthy foods, and a healthy choice of butter/margarine as a sandwich spread (low-fat percentage) was associated with overweight/obesity. Socioeconomic aspects of food habits could also be a piece of the puzzle to the reasons behind a dietary pattern because of high expenses for some food groups. However, over the last years, other alternatives to butter/margarine as a sandwich spread are offered on the market in Sweden, with a high-fat percentage but with a low proportion of saturated fat. It is also important to consider that young people's food choices are, to some extent, governed by what is offered at home and in school.

Former studies concerning the quality of food intake and body weight have shown diverging results^(7–9,21–25)^. In the present study, we found a statistically significant association between a high-frequency intake of vegetables and a lower proportion of overweight/obesity, in line with the inverse association between vegetable intake and weight among adults in a systematic review^(8)^. We found no statistically significant associations between a high-frequency intake of fruits or fish and isoBMI. A healthy choice of butter/margarine as a sandwich spread, as discussed before in the present paper, as well as a low-frequency intake of sweets/snacks, was associated with overweight/obesity. The inverse association between sweets/snacks and overweight has been seen before in a systematic review^(25)^. However, other results from this review showed different results. The associations between a reported healthy intake of some foods and overweight/obesity might be explained by a reverse causation so that being overweight or obese makes a person more conscious about food quality, while a thinner person could ‘afford’ to eat more sweets and snacks. Another possible explanation might be due to social desirability or underreporting of *unhealthy* foods, especially in obese people^(26)^. There is also a possibility that some foods may offer a greater satiety over other foods due to volume and energy density^(27)^, or affect appetite because of taste^(28)^, which, in turn, might have implications on food intake quantity. However, excess energy intake (food quantity regardless of food quality) is a major explanation for the development of overweight/obesity.

The diverging results in the present study where few choices of foods were associated with isoBMI go in line with other studies where it was concluded that both food quality and the total amount of food play an important role for health beyond weight^(29)^. In a meta-analysis, comparing different diets, there were no differences concerning the effect on weight reduction with respect to proportions of different macronutrients^(30)^. The weight reduction was equal for low-fat and low-carbohydrate diets. But when studying the association between the quality of food intake and mortality, all-cause mortality was higher with a low-carbohydrate diet^(31)^. To both promote health, achieve a healthy weight and reduce the risk for future non-communicable diseases and premature mortality, it is important to consider the quality of food as well as the total energy intake. The intake of single food groups may be associated with a healthy or unhealthy outcome, and INTERHEART, the world's largest case-control study of cardiac infarction, showed that consumption of fruits and vegetables at least once a day was associated with a lower risk of infarction^(32)^. In addition, a high intake of fruits and vegetables in childhood predicts a lower level of arteriosclerosis in adulthood, and adolescents with the highest fruit and vegetable consumption report the best health as adults^(33,34)^.

To stimulate the consumption of fruits and vegetables is important *per se*, as a high intake of fruits and vegetables is associated with good health, and as it perhaps may stimulate other healthy food choices.

### Strengths and limitations

The large study population from seven cohorts of 16-year-old adolescents is a strength of the study. Due to the cross-sectional design, we cannot say anything about causality. It is important to bear in mind that a cross-sectional study as this cannot explain if a person with a specific reported food intake or a body size recently has changed food choices, has lost, or gained weight or is weight stable. Neither have we considered other important determinants for weight development (e.g. physical activity, inactivity and metabolic regulation). The ‘My Health’ questionnaire mainly focuses on the quality of food intake and not on the energy intake, which, together with energy expenditure, are the key factors explaining weight^(35–38)^. The prevalence of overweight among girls in the present study is comparable with the results from another Swedish study, while there is a larger proportion of overweight among boys in the present study^(39)^. Collecting data with self-reported answers have limitations since there is a risk that the answers might not reflect the true dietary intake due to memory, psychological factors and social interaction as previously discussed in a study from partly the same study population^(3)^. However, this method is considered an acceptable method in large health surveys as in the Västerbotten Intervention Program^(40)^. In the present study, the questionnaire served as an underlay for a healthy dialogue, and by that, the reliability could be strengthened, since missing, unclear or unserious responses could be corrected at the health dialogue. The limited number of questions related to food intake in the ‘My Health’ questionnaire may restrict the possibilities of interpretation since it does not cover the whole dietary pattern. It might have been interesting to study the dietary intake in relation to background factors as for instance socioeconomic status, but due to the lack of such data, this was not possible. One could object to the categorisation of foods as in the questionnaire where a healthy intake of fruits includes no upper intake limit. This categorisation was due to the design of the answer options in the questionnaire. However, beside the high content of simple sugars in fresh fruit which could be pro-obesity, there are also possible anti-obesity mechanisms such as a high water and fibre content, a high inherent of micronutrients and non-essential phytochemicals, which could affect modulation in gut ecology^(7)^.

## Conclusions

Intakes of different healthy foods were mostly associated with each other in a large study group of 16-year-old adolescents during a 7-year period. A high-frequency intake of vegetables was associated with eating fruits and fish more often, and sweets/snacks and pastries less often.

A high-frequency intake of vegetables as well as a high-frequency intake of sweets/snacks and an unhealthy choice of butter/margarine were associated with a lower probability of overweight/obesity. The findings in this study increase the understanding of food habits among adolescents and emphasise the importance of taking the quality as well as the total amount of food into consideration when designing interventions targets both good health and a healthy weight.
